# Social Adversity Is Causally Linked to Multimorbidity Including Oral Conditions

**DOI:** 10.1177/00220345251362201

**Published:** 2025-09-05

**Authors:** A.O. Esemezie, D.J. Lizotte, G. Tsakos, N. Gomaa

**Affiliations:** 1 Dentistry, Schulich School of Medicine & Dentistry, Western University, London, ON, Canada; 2 Epidemiology & Biostatistics, Schulich School of Medicine & Dentistry, Western University, London, Ontario, Canada; 3 Computer Science, Faculty of Science, Western University, London, Canada; 4 Epidemiology & Public Health, University College London, London, UK

**Keywords:** aging, noncommunicable diseases, oral health, social capital, socioeconomic factors, causality

## Abstract

The fundamental cause theory posits social factors as causes of disease as they encompass access to important resources such as knowledge, wealth, and social networks. While these social factors have been consistently associated with oral and systemic diseases, causality remains unestablished. Here, we estimated the causal effect of social adversity, comprising low economic and social capital, on the development of (1) oral conditions (OC) and (2) multimorbidity including oral conditions (MIOC) in a cohort of middle-aged and older adults over a 7-y period and assessed whether effects varied by age or gender. We analyzed 2 waves from the Canadian Longitudinal Study on Aging (CLSA) (2011 and 2018). Social adversity comprised low economic (income) and social capital (community participation, social relationships). OC was defined as having 1 or more of poor self-reported oral health, lack of functional dentition (<20 natural teeth), or edentulism. Participants with an OC at baseline were excluded. MIOC was defined as having 2 or more chronic diseases and an OC. Logistic marginal structural models with inverse probability weighting estimated the causal odds ratio (OR) of developing both outcomes, controlling for sociodemographic and behavioral factors. In a total of 23,366 participants, 14% experienced social adversity at baseline, with a prevalence of 17% OC and 7% MIOC at follow-up. Social adversity significantly increased the odds of developing OC (OR = 1.9, 95% confidence interval [CI] 1.7, 2.2) and MIOC (OR = 1.7, 95% CI 1.5, 2.0) at follow-up. The observed effects were strongest in the middle-aged group, with similar odds observed in both men and women. Our findings indicate that social and economic capital are causally linked to the development of OC and MIOC over time. We suggest that policies for healthy aging should prioritize action on social and living conditions.

## Introduction

The proportion of older adults in Canada and globally is projected to increase in the coming decades, along with a consequent rise in the burden of noncommunicable chronic diseases, including oral diseases and multimorbidity (World Health Organization 2015). This demographic shift has led to global initiatives such as the United Nations–World Health Organization Collaborative on Healthy Aging to prompt investigations and action for optimizing the aging process beyond mere longevity to chronic disease prevention to support functionality and intrinsic capacity (World Health Organization 2020). Oral diseases are the most prevalent chronic conditions globally; thus, understanding their determinants has also been emphasized for the optimization of healthy aging (Colombo and Wu 2023; Rock et al. 2025).

Oral and systemic diseases share common determinants, with interconnected pathways that interact over the life course and that can become amplified with age (Weintraub et al. 2023). For example, while oral diseases are associated with diabetes, cardiovascular diseases, respiratory infections, and multimorbidity, through inflammatory pathways, among others (Bitencourt et al. 2023), age-related changes including compromised immunity, polypharmacy-induced xerostomia, inadequate oral hygiene due to physical or cognitive decline, affected nutrition, and stress can all exacerbate these oral–systemic disease connections around aging (Wu et al. 2023). However, traditional clinical interventions that target such biological and behavioral factors are arguably ineffective and unsustainable in preventing oral diseases and related chronic conditions (Gomaa et al. 2019; McGrath et al. 2009). Notably, social adversity including lower income and education, poor social networks and social participation, and limited access to oral health care around retirement can also aggravate the risk to health in older age (Aida et al. 2022). These key aspects of social adversity have been theorized as causes of disease by Link and Phelan’s fundamental cause theory, which posits modifiable factors such as economic (income, wealth, and access to resources) and social capital (resources embedded in social networks) (Bourdieu 1986) to be at the root of health by influencing one’s exposure to risks, related aggravating or protective mechanisms, and their access to enabling or disabling factors (Link and Phelan 1995). The theory suggests that while the behavioral and biological mechanisms underlying disease and morbidity evolve, resources continue to affect health by allowing individuals greater access to resources, thus positioning them to avoid health risks and adapt to adverse changes. Consequently, addressing deficiencies in such resources through tackling the fundamental social conditions that put individuals at “the risk of risks of disease” in the first place is crucial for prevention. Arguably, interventions that target only disease-specific downstream factors are likely to have limited impact on reducing health inequalities and may even widen the social gap in health.

Despite the robust epidemiological evidence linking economic and social capital to oral and systemic health outcomes, causality has not yet been established (Schuch et al. 2023). Traditional association studies provide valuable insights but are unable to disentangle the complex relationships between these social factors and health outcomes, thereby limiting their ability to conclusively inform interventions. This is due to unmeasured confounding, possibility of reverse causality, and lack of temporality (Listl et al. 2022). Causal inference modeling has been suggested to overcome these limitations by accounting for time-varying confounding to better approximate causal effects (Hernán and Robins 2023).

Thus, the objective of this study was to estimate the total causal effect of social adversity, defined as lower economic and social capital, on the development of oral conditions (OC) and multimorbidity including oral conditions (MIOC) over time, in a cohort of middle-aged and older Canadians. The impact of social adversity can differentially affect age groups. As well, although men reportedly have worse oral health, women typically experience greater social inequalities than men do and have been consistently shown to be more vulnerable to their health impacts due to intersecting social and economic roles across the life course (Limo et al. 2024). We therefore further examined whether any causal effects varied by age and gender.

## Methods

### Data Source and Study Population

**Figure 1.**
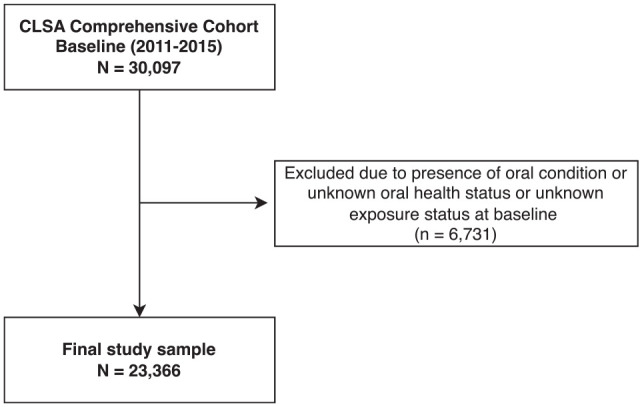
Participant flow diagram demonstrating the analytical sample.

We used data from 2 waves of the Comprehensive Cohort of the Canadian Longitudinal Study on Aging (CLSA): baseline (2011–2015) and second follow-up (2018–2021). The CLSA is a longitudinal study of participants aged 45 to 85 y at the time of recruitment sampled from all 10 Canadian provinces, excluding Indigenous people living on reserves, institutionalized individuals, and full-time members of the Canadian military (Raina et al. 2019). We excluded participants who reported an OC or had missing data on either OC status or social adversity at baseline (*n* = 6,731). Our analytical sample comprised 23,366 participants (Fig. 1).

### Outcome Variables

Oral health and chronic disease outcomes were self-reported at the second CLSA follow-up. OC was defined as the presence of at least 1 of the following: poor self-reported oral health (SROH), lack of functional dentition (<20 natural teeth) (WHO Expert Committee 1992), or edentulism. This allowed us to capture various aspects of oral health. MIOC was defined as the presence of at least 2 chronic diseases (multimorbidity) and an OC. Following the Public Health Agency of Canada definition, we operationalized multimorbidity as having 2 or more of cardiovascular disease, cancer, arthritis, respiratory disease, neurologic disease, diabetes, and mental health disorders (Roberts et al. 2015).

### Exposure Variable

Social adversity was a composite variable of low economic capital and low social capital. Low economic capital was defined as having a household income of less than $50,000 per annum (Statistics Canada 2019). Social capital, which refers to social resources that are available to an individual and their community through social relationships (Bourdieu 1986), was categorized into structural (measure of social activity and networks) and cognitive social capital (measure of social support and cohesion) (Moore and Kawachi 2017). Structural social capital was measured using a Likert-type scale ranging from 1 to 4 from the social participation module assessing the frequency of community-related activity: 1 (*yearly*), 2 (*monthly*), 3 (*weekly*), and 4 (*daily*). Cognitive social capital was calculated from the 19 questions on the Medical Outcomes Study–Social Support Survey, which measures perceived support across 4 domains and generates a 0 to 100 overall score, with higher values indicating greater support (Sherbourne and Stewart 1991). Participants were categorized as having low social capital if they had lower scores for structural (0 to 2) or cognitive (<82.5) social capital (Hensel and Gomaa 2023).

### Confounding Variables

**Figure 2.**
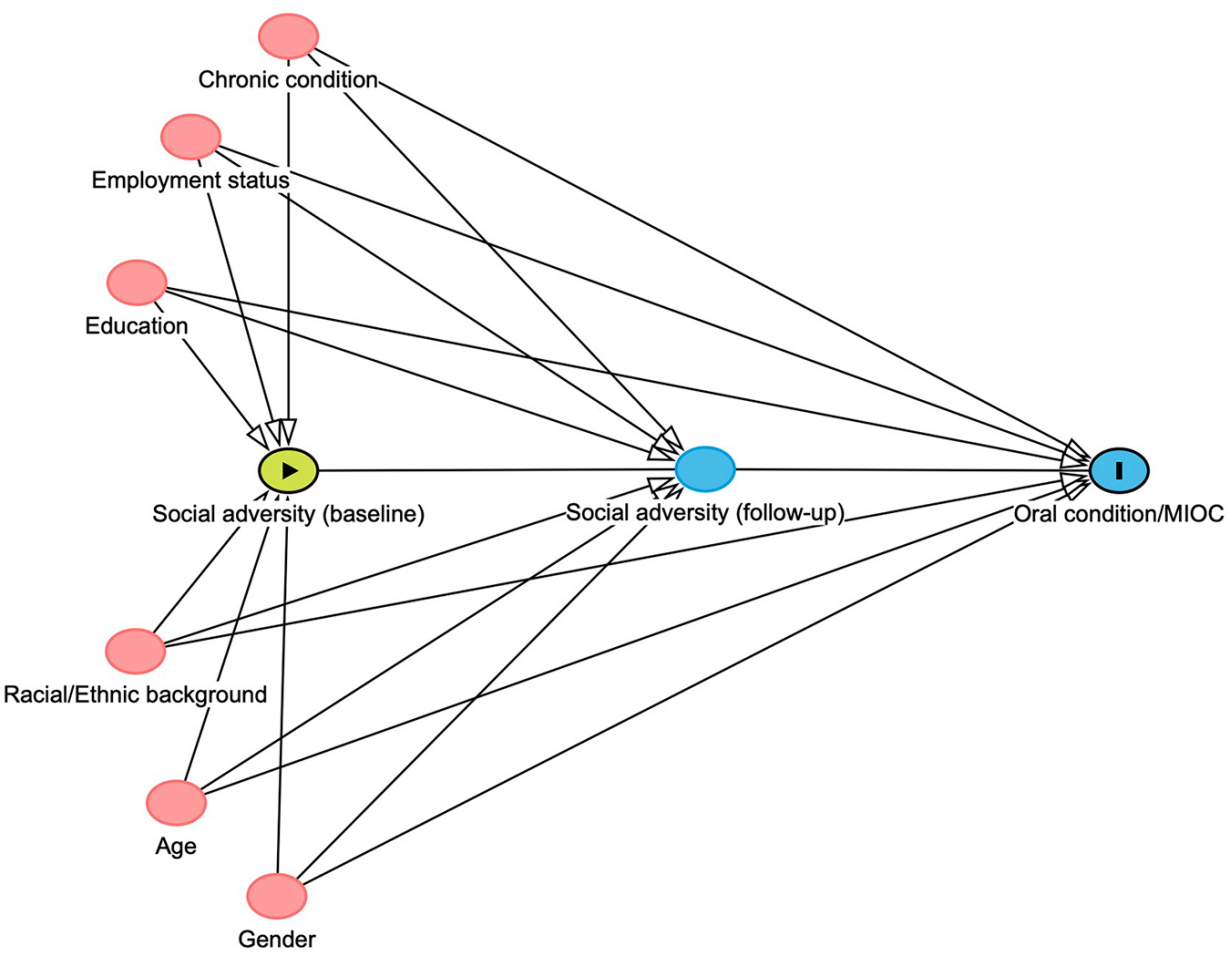
Directed acyclic graph (DAG) for the exposure–outcome relationship constructed using DAGitty 3.1. Data for the exposure (social adversity) were assessed at baseline and at follow-up 2. Baseline confounding variables are indicated with a red node.

These were selected a priori based on theory and literature (Fig. 2). Variables initially identified as confounders included age, gender, race/ethnicity, level of education, employment status, and the presence of chronic disease, to account for preexisting health status and minimize bias from baseline morbidity. Smoking and alcohol consumption lie on the causal pathway between social adversity and health outcomes. While controlling for mediators may lead to overestimation bias (Schisterman et al. 2009), we additionally adjusted for these behaviors to test for their effect on the observed causal estimates.

### Statistical Analysis

To assess the causal effects of social adversity on each of OC and MIOC at follow-up, we fitted a logistic marginal structural model (MSM). Using inverse probability weighting (IPW), stabilized treatment weights were constructed to account for time-varying exposures, confounding and censoring. Social adversity was modeled as a time-varying exposure, measured at both baseline and follow-up, while employment status was modeled as a time-varying confounder. Other covariates were treated as fixed based on baseline values. Stabilized weights for each participant were calculated as the ratio of the marginal probability of experiencing social adversity at each time point (numerator) to the conditional probability of experiencing social adversity given both baseline and time-varying covariates (denominator) of that time point. These probabilities were estimated using logistic regression models. To reduce the bias arising from attrition and loss to follow-up, stabilized censoring weights were calculated using a logistic model. Stabilized time point–specific treatment weights and censoring weights were then multiplied to generate an overall final weight for each participant, creating a pseudo-population without covariate imbalance. With this pseudo-population, the causal odds ratio (OR) could then be estimated by fitting a weighted logistic MSM. To explore subgroup differences, we stratified our analysis by age group and gender. Missing data were handled by generating 20 imputations using the multivariate imputation by chained equations (mice) package in R. All statistical analyses were conducted using R statistical software (version 4.4.2).

### Sensitivity Analysis

E-values assess how strongly an unmeasured confounder would need to be associated with both exposure and outcome to explain the observed effects (VanderWeele and Ding 2017). In addition, to assess the influence of selection bias due to censoring (e.g., mortality, loss to follow-up), we compared the baseline characteristics of participants in the second follow-up to those who did not participate in the second follow-up in our sample. To further examine the sensitivity of the MIOC variable, we used an alternate threshold of having 1 or more chronic conditions with an OC. Lastly, we explored the independent effects of economic and social capital by disaggregating these exposures and examining their separate effects on OC and MIOC.

### Ethical Considerations and Reporting Guidelines

This project received approvals from the Human Research Ethics Board, Western University (project ID 120939) and the CLSA for data access (application ID 2401024) and conforms to the STROBE guidelines (Appendix).

## Results

### Characteristics of the Study Population

Among the total of 23,366 participants, 14% (*n* = 3,304) had experienced social adversity at baseline (Table 1). Among these, 61% were women, 94% were of White race/ethnicity, 24% were older than 75 y, 70% were retired, and 75% reported being diagnosed with at least 1 chronic condition. At the 7-y follow-up, 17% of the total sample had an OC and 7% had MIOC. Among those who were exposed to social adversity at baseline, 31% had an OC at follow-up. Of those with an OC in this group, 35% reported poor SROH, 73% lacked functional dentition, and 6% were edentulous. In addition, 14% of participants exposed to social adversity reported having MIOC at follow-up.

**Table 1.** Characteristics of Study Participants Stratified by Baseline Exposure to Social Adversity.

	Full Sample (*N* = 23,366)	Social Adversity (*n* = 3,304)	No Social Adversity (*n* = 20,062)	*P* Value^a^
Baseline characteristics				
Gender, *n* (%)				
Men	111,657 (50)	1,281 (39)	10,376 (52)	<0.001
Women	11,709 (50)	2,023 (61)	9,686 (48)	
Racial/ethnic background, *n* (%)				
White	22,440 (96)	3,108 (94)	19,332 (96)	<0.001
Non-White^b^	926 (4)	196 (6)	730 (4)	
Age group at baseline, *n* (%)				
45–54	6,435 (28)	494 (15)	5,941 (30)	<0.001
55–64	8,030 (34)	1,014 (31)	7,016 (35)	
65–74	5,511 (24)	993 (30)	4,518 (23)	
75–85	3,390 (15)	803 (24)	2,587 (13)	
Education level attained at baseline, *n* (%)				
Postsecondary degree/diploma	18,836 (81)	2,206 (67)	16,630 (83)	<0.001
Some postsecondary education	1,661 (7)	338 (10)	1,323 (7)	
Secondary school graduation, no postsecondary education	1,991 (9)	419 (13)	1,572 (8)	
Less than secondary school graduation	851 (3)	331 (10)	520 (2)	
Missing	27	10	17	
Employment status at baseline, *n* (%)				
Employed	10,040 (43)	759 (23)	9,281 (46)	<0.001
Unemployed	983 (4)	240 (7)	743 (4)	
Retired	12,339 (53)	2,304 (70)	10,035 (50)	
Missing	4	1	3	
Baseline, annual household income, *n* (%)				
<$20,000	848 (4)	643 (19)	205 (1)	<0.001
$20,000–$49,999	4,619 (20)	2,661 (81)	1,958 (10)	
$50,000–$99,999	8,488 (36)	—	8,488 (42)	
$100,000–$149,999	4,950 (21)	—	4,950 (25)	
≥$150,000	4,461 (19)	—-	4,461 (22)	
Low cognitive social capital, *n* (%)				
Yes	9,154 (39)	2,996 (92)	6,158 (31)	<0.001
No	14,135 (61)	262 (8)	13,873 (69)	
Missing	77	46	31	
Low structural social capital, *n* (%)				
Yes	3,106 (13)	870 (26)	2,236 (11)	<0.001
No	20,259 (87)	2,433 (74)	17,826 (89)	
Missing	1	1	0	
Presence of a chronic condition at baseline, *n* (%)				
Yes	14,931 (64)	2,467 (75)	12,464 (62)	<0.001
No	8,435 (36)	837 (25)	7,598 (38)	
Prevalence of chronic conditions at baseline, *n* (%)				
Arthritis (including osteoarthritis and rheumatoid arthritis)	6,253 (27)	1,103 (33)	5,150 (26)	-
Anxiety and/or mood disorders	4,656 (20)	976 (30)	3,680 (18)	
Diabetes	3,781 (16)	713 (22)	3,068 (15)	
Respiratory disease (asthma and/or chronic obstructive pulmonary disease)	3,738 (16)	630 (19)	3,108 (15)	
Cancer	3,438 (15)	532 (16)	2,906 (14)	
Cardiovascular disease (heart disease and/or stroke)	2,617 (11)	506 (15)	2,111 (11)	
Neurologic disease (Alzheimer’s disease or related dementias)	36 (0)	6 (0)	30 (0)	
Presence of multimorbidity at baseline, *n* (%)				
Yes	6,739 (29)	1,308 (40)	5,431 (27)	<0.001
No	16,627 (71)	1,996 (60)	14,631 (73)	
Follow-up characteristics				
Social adversity at follow-up, *n* (%)				
Yes	2,669 (14)	1,463 (64)	1,206 (7)	<0.001
No	16,368 (86)	840 (36)	15,528 (93)	
Missing	4,329	1,001	3,328	
Employment status at follow-up, *n* (%)				
Employed	8,030 (38)	632 (23)	7,398 (41)	<0.001
Unemployed	936 (5)	133 (5)	803 (4)	
Retired	11,894 (57)	1,948 (72)	9,946 (55)	
Missing	2,506	591	1,915	
Presence of OC at follow-up, *n* (%)				
Yes	3,417 (17)	807 (31)	2,610 (15)	<0.001
Poor SROH alone	752 (22)	172 (21)	580 (22)	
Poor SROH and edentulism	25 (0)	11 (1)	14 (0)	
Poor SROH and lack of functional dentition	331 (10)	102 (13)	229 (9)	
Lack of functional dentition alone	2,145 (63)	482 (60)	1,663 (64)	
Edentulism alone	164 (5)	40 (5)	124 (5)	
No	17,008 (83)	1,805 (69)	15,203 (85)	
Missing	2,941	692	2,249	
Presence of MIOC at follow-up, *n* (%)				
Yes	1,387 (7)	367 (14)	1,020 (6)	<0.001
No	19,038 (93)	2,245 (86)	16,793 (94)	
Missing	2,941	692	2,249	

MIOC, multimorbidity inclusive of oral conditions; OC, oral conditions; SROH, self-reported oral health.

a *P* values were based on the chi-square test between exposure groups.

b Among participants categorized as “non-White” (*n* = 926), the most commonly reported racial/cultural backgrounds were those identifying with multiple origins (38%), South Asian (22%), Chinese (16%) and Black (17%). Other groups including Arab, Korean, Southeast Asian, Latin American, and others each represented less than 10% of the non-White category. Due to small subgroup sizes, these were not analyzed separately.

### Causal Effect of Social Adversity on Developing OC and MIOC

IPW and weighted MSM showed that participants who had experienced social adversity at baseline had twice the odds of developing an OC (OR = 1.9, 95% CI 1.7, 2.2) and MIOC (OR = 1.7, 95% CI 1.5, 2.0) over the 7-y period compared with those who did not experience social adversity. These results were consistent in crude and adjusted models **(**Table 2). Adjusting for smoking status and alcohol use by incorporating these variables into the inverse probability weights resulted in slightly attenuated effect estimates for OC (OR = 1.7, 95% CI 1.5, 2.0) and MIOC (OR = 1.6, 95% CI 1.4, 1.8), indicating that while these behaviors may account for part of the association, a substantial effect on both outcomes persists. Experiencing low economic capital alone (without low social capital) increased the odds for both OC (OR = 1.8, 95% CI 1.6, 2.0) and MIOC (OR = 1.6, 95% CI 1.5, 1.9). Low cognitive social capital was also associated with increased odds for OC (OR = 1.3, 95% CI 1.2, 1.4) and MIOC (OR = 1.2, 95% CI 1.0, 1.4), with similar effects observed for low structural social capital.

**Table 2.** Results of Crude and Weighted Marginal Structural Models (MSM) Showing Estimates of the Causal Relationship between Social Adversity and the Development of Oral Conditions (OC) and Multimorbidity Inclusive of Oral Conditions (MIOC) at Follow-up.

	OC	MIOC
	OR (95% CI)	OR (95% CI)
Social adversity (low economic and social capital)		
Crude effect^a^	2.6 (2.2, 2.9)	2.4 (2.1, 2.7)
Total effect^b^	1.9 (1.7, 2.2)	1.7 (1.5, 2.0)
Total effect^c^	1.7 (1.5, 2.0)	1.6 (1.4, 1.8)
Low economic capital		
Crude effect^a^	2.6 (2.3, 2.9)	2.5 (2.2, 2.8)
Total effect^b^	1.8 (1.6, 2.0)	1.6 (1.5, 1.9)
Total effect^c^	1.6 (1.4, 1.8)	1.5 (1.3, 1.7)
Low cognitive social capital		
Crude effect^a^	1.5 (1.3, 1.6)	1.4 (1.3, 1.6)
Total effect^b^	1.3 (1.2, 1.4)	1.2 (1.0, 1.4)
Total effect^c^	1.2 (1.1, 1.4)	1.2 (1.0, 1.3)
Low structural social capital		
Crude effect^a^	1.3 (1.1, 1.4)	1.2 (1.1, 1.4)
Total effect^b^	1.2 (1.1, 1.4)	1.2 (1.1, 1.4)
Total effect^c^	1.2 (1.0, 1.3)	1.1 (1.0, 1.3)

CI, confidence interval; OR, odds ratio.

a Crude unadjusted effect.

b MSM with inverse probability weighting to adjust for age, gender, race/ethnicity, employment status at baseline and follow-up, education level, and baseline presence of chronic conditions. The weights account for exposure at both baseline and follow-up as well as potential censoring.

c MSM with inverse probability weighting to adjust for age, gender, race/ethnicity, employment status at baseline and follow-up, education level, baseline presence of chronic conditions, smoking status, and alcohol use. The weights account for exposure at both baseline and follow-up as well as potential censoring.

Age- and gender-stratified MSM revealed that social adversity affected the development of OC and MIOC at follow-up across age groups and in both men and women (Table 3). The greatest effect magnitude was observed among individuals aged 45 to 54 y, with the odds of developing OC (OR = 2.2, 95% CI 1.6, 3.0) being higher than for MIOC (OR = 2.0, 95% CI 1.4, 3.0) in this age group. The effects of social adversity were of less magnitude in the older age group. Sex-stratified models demonstrated significant effects of social adversity on the development of OC and MIOC at follow-up in both genders, with a similar magnitude of effects observed in both men and women for OC and MIOC.

**Table 3.** Results of Age- and Gender-Stratified Marginal Structural Models (MSM) Showing Estimates of the Causal Relationship between Social Adversity and the Development of Oral Conditions (OC) and Multimorbidity Inclusive of Oral Conditions (MIOC) at Follow-up.

	Oral Condition	MIOC
	OR (95% CI)	OR (95% CI)
Age stratified^a^		
45 to 54 y	2.2 (1.6, 3.0)	2.0 (1.4, 3.0)
55 to 64 y	2.0 (1.7, 2.5)	1.8 (1.4, 2.3)
65 to 74 y	1.8 (1.5, 2.1)	1.6 (1.3, 2.0)
75+ y	1.4 (1.2, 1.8)	1.3 (1.0, 1.7)
Gender stratified^a^		
Men	1.9 (1.6, 2.2)	1.7 (1.4, 2.1)
Women	1.9 (1.6, 2.2)	1.8 (1.5, 2.1)

CI, confidence interval; OR, odds ratio.

a MSM with inverse probability weighting to adjust for age, gender, race/ethnicity, employment status at baseline and follow-up, education level, and baseline presence of chronic conditions. The weights account for exposure to social adversity at both baseline and follow-up as well as potential censoring.

### Results of Sensitivity Analysis

The comparison of baseline characteristics showed that older participants, particularly those aged 65 to 85 y, were less likely to participate in the second follow-up, suggesting greater loss to follow-up in older age groups. Nonparticipants also included a higher proportion of individuals with social adversity and chronic conditions (Appendix Table S1). The E-values for the total causal effect of social adversity on having an OC and MIOC were 2.1 and 2.8, respectively, suggesting that a moderate amount of unmeasured confounding is required to fully explain the observed effects (Appendix Table S2). For the age-stratified analysis, the E-values for having an OC and MIOC among individuals aged 45 to 54 y and 55 to 64 y were all >2, indicating moderately robust estimates. Similarly, when stratified by gender, the causal effects for developing the outcomes remained robust, with E-values >2 for both men and women. When using an alternate definition for MIOC, its prevalence increased to 12%; however, the causal effect of social adversity on developing MIOC at follow-up remained relatively similar (OR = 1.8, 95% CI 1.6, 2.1) (Appendix Table S3).

## Discussion

We demonstrate a causal relationship between social adversity and the development of OC and MIOC in a cohort of middle-aged and older adults. Using 2 waves of CLSA and applying causal inference models, our results showed that those experiencing social adversity had twice the odds of developing these outcomes over a 7-y period, adjusted for confounders. Stratification by age revealed the effect of social adversity to be strongest in the middle-aged group (45–54 y). Our estimates represent the average effect of the observed combination of low economic capital and low social capital as they naturally co-occurred in the study population rather than the effect of a specific, well-defined intervention.

Our findings are consistent with previous research on the common risk factors for oral and systemic diseases (Sheiham and Watt 2000). While prior studies investigated the impact of early-life adversity on later health outcomes, our analysis focused on social adversity experienced in mid to late adulthood. For instance, a study using MSMs found that early-life socioeconomic status was significantly associated with developing coronary heart disease, diabetes, and stroke in adulthood, although these effects were attenuated after adjusting for adult socioeconomic status (Nandi et al. 2012). Another study demonstrated that lower income had a causal effect on experiencing preventable hospitalizations and comorbidities (Cho et al. 2019). More recently, Mirza et al. (2024) showed the relationship between social disadvantage and multimorbidity to significantly increase when including those with OC.

The observed causal effects between social adversity and OC and MIOC may be mediated by an individual’s behaviors, access to resources, and biopsychosocial factors, all of which may be further amplified by aging (Gomaa 2022). In stratified analyses, we found the effect of social adversity to be consistent across all age categories but most pronounced in middle-aged adults (45–54 y), with reduced effects in individuals aged 75 y and older. This was consistent with a previous study that examined multimorbidity across age groups (St Sauver et al. 2015) showing that while the incidence of multimorbidity increased in older age, the absolute number of people with incident multimorbidity was substantially higher in the middle-aged groups. Similar to previous studies, this emphasizes the importance of early intervention to mitigate the cumulative effects in later life (Hensel et al. 2025), especially that poor oral health can also lead to reduced social participation, possibly exacerbating social adversity (Cooray et al. 2023).

We also found the magnitude of the effect of social adversity to be similar between men and women. Previous CLSA studies have reported that women had a higher prevalence of multimorbidity across 4 different definitions compared with men (Im et al. 2022). Women also generally have better oral health and oral health behaviors including regular dental visits, preventive care, and healthier lifestyles than men do (Su et al. 2022). However, in our study, most individuals experiencing social adversity were women (61%). While our study does not explore the specific mechanisms underlying these inequalities, our results are consistent with previous Canadian findings showing both men and women living in social adversity to be at risk of poor oral health (Canadian Academy of Health Sciences 2014; Limo et al. 2024).

### Strengths and Limitations

A key strength of our study is the application of causal inference models. Assessing the causal impacts of social exposures is often neither ethical nor feasible, making it challenging to establish causal effects through traditional methods (Brocklehurst et al. 2019). Another strength is the use of a large, population-based longitudinal cohort study and the inclusion of OC into the overall multimorbidity definition. Our study is not without limitations. While MSMs can estimate causal effects, this relies on several assumptions, including consistency. Consistency can be particularly challenging with complex exposures (Kaufman 2019) such as social adversity, which combines low economic and low social capital. Different interventions targeting these components may produce varying effects on oral and systemic health. However, additional analyses examining each component separately showed that low income had the strongest association with outcomes, with structural and cognitive social capital also contributing significantly, although with a smaller effect size. This suggests that the composite exposure captures key dimensions of social adversity in this population.

Despite using IPW to balance for known confounders, the possibility of unmeasured confounders could not be ruled out, possibly violating the exchangeability assumption (Hernán and Robins 2023). In addition, reliance on self-reported data may introduce measurement bias. However, self-reported oral health measures have been validated and are widely employed in epidemiological studies (Matsui et al. 2016). Although the CLSA was broadly inclusive of community-dwelling adults aged 45 y and older, the CLSA sample consisted predominantly of well-educated, White participants. Consequently, despite the significant effect we observed between social adversity and oral/systemic health outcomes in this sample, it may still be an underestimation of what would be observed in more socioeconomically and racially/ethnically diverse populations (Fleming et al. 2023).

Future research building on this work can incorporate more nuanced longitudinal assessments of oral health trajectories. While our study assessed incident OC, this approach does not account for the complexity of oral health dynamics (i.e., recovery or deterioration), as the use of composite and primarily categorical variables in the current study limited the feasibility of modeling graded associations. Capturing such variations would require more frequent measurement points and the utilization of continuous or count-based oral health measures such as probing depths or the number of decayed teeth. Such investigations could provide deeper insight into how social adversity shapes oral health across the life course and potentially help identify windows for intervention.

### Public Health Policy Implications

Chronic diseases remain highly prevalent worldwide with a substantial individual and societal burden (GBD 2019 Risk Factors Collaborators 2020). These are projected to increase in the coming decades due to the global aging population. Our findings are in alignment with Link and Phelan’s fundamental cause theory, which posited social adversity as causes of disease and called for upstream policies that targeted these social factors for effective disease prevention (Link and Phelan 1995). Public health policies that aim to improve social and economic capital such as improving financial security and enhancing social support systems are important for mitigating the burden of chronic diseases in aging populations. Implementing life course interventions early on may help prevent the accumulation of risk factors that lead to adverse outcomes in middle-age and later life.

## Conclusion

Social adversity, including low social and economic capital, has a causal effect on the development and the accumulation of chronic diseases over time. While these effects are observed across the board, they are particularly pronounced in middle-aged adults. With the growing burden of chronic diseases in Canada and globally and the rise in calls for prioritizing aging populations on national and international fronts, upstream interventions that tackle the social determinants of health and that improve social and living conditions will be key for healthy aging.

## Author Contributions

A.O. Esemezie, contributed to conception, design, data acquisition, analysis, and interpretation, drafted and critically revised the manuscript; D.J. Lizotte, G. Tsakos, contributed to data interpretation, critically revised the manuscript; N. Gomaa, contributed to conception, design, data acquisition and interpretation, drafted and critically revised the manuscript. All authors gave final approval and agree to be accountable for all aspects of the work.

## Supplemental Material

sj-doc-1-jdr-10.1177_00220345251362201 – Supplemental material for Social Adversity Is Causally Linked to Multimorbidity Including Oral Conditions Supplemental material, sj-doc-1-jdr-10.1177_00220345251362201 for Social Adversity Is Causally Linked to Multimorbidity Including Oral Conditions by A.O. Esemezie, D.J. Lizotte, G. Tsakos and N. Gomaa in Journal of Dental Research

sj-docx-1-jdr-10.1177_00220345251362201 – Supplemental material for Social Adversity Is Causally Linked to Multimorbidity Including Oral Conditions Supplemental material, sj-docx-1-jdr-10.1177_00220345251362201 for Social Adversity Is Causally Linked to Multimorbidity Including Oral Conditions by A.O. Esemezie, D.J. Lizotte, G. Tsakos and N. Gomaa in Journal of Dental Research
