# Supplementary material for: Social Adversity Is Causally Linked to Multimorbidity Including Oral Conditions
Source: J Dent Res. 2025 Sep 5;105(1):103–11. doi: 10.1177/00220345251362201 (PMC12701912; doi:10.1177/00220345251362201)
Supplement: sj-docx-1-jdr-10.1177_00220345251362201 – Supplemental material for Social Adversity Is Causally Linked to Multimorbidity Including Oral Conditions [file sj-docx-1-jdr-10.1177_00220345251362201.docx]

**Supplementary file**

**Social Adversity is Causally Linked to Multimorbidity Including Oral Conditions**

Alex O. Esemezie^1,2^, Daniel J. Lizotte^2,3^, Georgios Tsakos^4^, Noha A. Gomaa^1,2^

1. Dentistry, Schulich School of Medicine & Dentistry, Western University, London, ON, Canada
2. Epidemiology & Biostatistics, Schulich School of Medicine & Dentistry, Western University, London, Ontario, Canada
3. Computer Science, Faculty of Science, Western University, London, Canada
4. Epidemiology & Public Health, University College London, London, United Kingdom

**Variables**

- Age (grouped into four categories of 45-54, 55-64, 65-74, 75-85 years)
- Gender (men, women)
- Race/Ethnicity (white, non-white): Race/ethnicity was derived from self-reported responses in the CLSA baseline data. Due to small sample sizes across several racialized groups, and to preserve statistical power and model stability, we categorized race/ethnicity into two broad groups: White and Non-White. While this dichotomization is a common analytic strategy in large-scale epidemiologic studies, we acknowledge that it limits the ability to explore important differences between specific racial/ethnic communities.
- Level of education (post-secondary education, some post-secondary education, secondary school graduation but no post-secondary education, less than secondary school graduation)
- Employment status (employed, retired, unemployed)
- Presence of one chronic disease at baseline (yes, no)
- Smoking status (current, former, never)
- Alcohol use (regular drinker, occasional drinker, did not drink in the last 12 months)
- Self-Reported Oral Health (SROH): SROH was measured using a single-item Likert response to the question: “In general, would you rate the health of your mouth as excellent, very good, good, fair, or poor?”. This was then dichotomized into poor (fair or poor) and good SROH (excellent, very good, good)
- Functional dentition (< 20 natural teeth): Functional dentition was defined based on the response to the question: “Do you have 20 or more natural teeth?”.
- Edentulism: Before assessing functional dentition, individuals were asked about their edentulism status, through the question: “Do you have one or more of your own original teeth?”. Only those who were not identified as edentulous were subsequently asked about their functional dentition, preventing variable overlap.
- Chronic conditions: Chronic conditions were self-reported through the question: “Has a doctor ever told you that you have ….?”.
  - Cardiovascular disease (heart disease and/or stroke)
  - Cancer
  - Arthritis (including osteoarthritis and/or rheumatoid arthritis)
  - Respiratory disease (chronic obstructive pulmonary disease and/or asthma)
  - Neurological disease (Alzheimer’s disease or related dementias)
  - Diabetes
  - Mental health disorder (anxiety and/or mood disorder)

**Table S1.** Comparison of baseline characteristics between participants who did and did not participate in the second follow-up of the study sample.

|  | **Did not participate in second follow-up**  **(n = 2502)** | **Participated in second follow-up**  **(n = 20864)** | **p-value** |
| --- | --- | --- | --- |
| **Gender, N (%)** |  |  |  |
| Men | 1318 (53) | 10339 (50) | 0.003 |
| Women | 1184 (47) | 10525 (50) |  |
|  |  |  |  |
| **Racial/ethnic background, N (%)** |  |  |  |
| White | 2351 (94) | 20089 (96) | <0.001 |
| Non-White | 151 (6) | 775 (4) |  |
|  |  |  |  |
| **Age group at baseline, N (%)** |  |  |  |
| 45-54y | 475 (19) | 5960 (29) | <0.001 |
| 55-64y | 557 (22) | 7473 (36) |  |
| 65-74y | 652 (26) | 4859 (23) |  |
| 75-85y | 818 (23) | 2572 (12) |  |
|  |  |  |  |
| **Education level attained at baseline, N (%)** |  |  |  |
| Post-secondary degree/diploma | 1834 (74) | 17002 (82) | <0.001 |
| Some post-secondary education | 213 (9) | 1448 (7) |  |
| Secondary school graduation, no post-secondary education | 278 (11) | 1713 (8) |  |
| Less than secondary school graduation | 170 (7) | 681 (3) |  |
| Missing | 7 | 20 |  |
|  |  |  |  |
| **Employment status at baseline, N (%)** |  |  |  |
| Employed | 714 (29) | 9326 (45) | <0.001 |
| Unemployed | 131 (5) | 852 (4) |  |
| Retired | 1657 (66) | 10682 (51) |  |
| Missing | 0 | 4 |  |
|  |  |  |  |
| **Social adversity exposure at baseline, N (%)** |  |  |  |
| Yes | 590 (24) | 2714 (13) | <0.001 |
| No | 1912 (76) | 18150 (87) |  |
|  |  |  |  |
| **Presence of > 1 chronic condition at baseline, N (%)** |  |  |  |
| Yes | 1807 (72) | 13124 (63) | <0.001 |
| No | 695 (28) | 7740 (37) |  |

P-values based on Chi-square test between groups.

**Table S2.** E-value sensitivity analysis for effect estimate parameters

| **Parameter** | **Oral condition** | **MIOC** |
| --- | --- | --- |
|  | **E-value**  **(lower limit)** | **E-value**  **(lower limit)** |
| **Total causal effect** | 2.1 (1.9) | 2.8 (2.4) |
| **Age-stratified** | | |
| **45 to 54y** | 2.4 (1.8) | 3.4 (2.2) |
| **55 to 64y** | 2.2 (1.9) | 3.0 (2.2) |
| **65 to 74y** | 2.0 (1.8) | 2.6 (1.9) |
| **75y+** | 1.7 (1.4) | 1.9 (1.0) |
| **Gender-stratified** | | |
| **Men** | 2.1 (1.8) | 2.8 (2.2) |
| **Women** | 2.1 (1.8) | 3.0 (2.4) |

**Table S3.** Results of Marginal Structural Models (MSM) showing estimates of the causal relationship between social adversity and the development of Multimorbidity Inclusive of Oral Conditions 1+ (MIOC1+) at follow-up.

|  | **MIOC1+** |
| --- | --- |
|  | **Odds Ratio**  **(95% CI)** |
| **Total causal effect^a^** | 1.8  (1.6, 2.1) |
| **Age-stratified** | |
| **45 to 54y** | 2.0  (1.4, 2.9) |
| **55 to 64y** | 2.0  (1.6, 2.5) |
| **65 to 74y** | 1.7  (1.5, 2.1) |
| **75y+** | 1.4  (1.2, 1.8) |
| **Gender-stratified** | |
| **Men** | 1.8  (1.5, 2.1) |
| **Women** | 1.9  (1.6, 2.2) |

^a^ MSM with inverse probability weighting to adjust for age, gender, race/ethnicity, employment status at baseline and follow-up, education level and baseline presence of chronic conditions. The weights account for exposure to social adversity at both baseline and follow-up, as well as potential censoring.

The prevalence of MIOC1+ in the sample at follow-up was 12%, with a higher prevalence in the social adversity group (25%) to those in the unexposed group (11%)

**Figure S1.** Analytic sample after excluding individuals who did not participant in second follow-up

**Table S4.** Results of crude and adjusted Marginal Structural Models (MSM) showing estimates of the causal relationship between social adversity and the development of OC and MIOC at follow-up after excluding all individuals who did not participate in second follow-up.

|  | **Oral condition** | **MIOC** |
| --- | --- | --- |
|  | **OR (95% CI)** | **OR (95% CI)** |
| **Total effect^a^** | 2.6  (2.3, 2.8) | 2.7  (2.4, 3.1) |
| **Total effect^b^** | 2.0  (1.8, 2.2) | 2.0  (1.7, 2.3) |

^a^ MSM with crude, unadjusted effects.

^b^ MSM with inverse probability weighting age, gender, race/ethnicity, employment status at baseline and follow-up, education level and baseline presence of chronic conditions.

**Table S5**. Results of age and gender-stratified Marginal Structural Models (MSM) showing estimates of the causal relationship between social adversity and the development of OC and MIOC at follow-up after excluding all individuals who did not participate in second follow-up.

|  | **Oral condition** | **MIOC** |
| --- | --- | --- |
|  | **OR (95% CI)** | **OR (95% CI)** |
| **Age-stratified**^a^ |  |  |
| **45 to 54y** | 2.5  (1.8, 3.4) | 3.9  (2.6, 6.0) |
| **55 to 64y** | 2.4  (2.0, 2.9) | 2.3  (1.7, 3.0) |
| **65 to 74y** | 1.7  (1.4, 2.1) | 1.6  (1.3, 2.0) |
| **75y+** | 1.3  (1.1, 1.6) | 1.1  (0.9, 1.5) |
| **Gender-stratified**^b^ |  |  |
| **Men** | 1.9  (1.6, 2.2) | 1.7  (1.4, 2.2) |
| **Women** | 2.1  (1.8, 2.4) | 2.2  (1.8, 2.7) |

^b^ MSM with inverse probability weighting adjusted for age, race/ethnicity, employment status at baseline and follow-up, education level and baseline presence of chronic conditions.

STROBE Statement—Checklist of items that should be included in reports of ***cohort studies***

|  | Item No | Recommendation | Page No. |
| --- | --- | --- | --- |
| **Title and abstract** | 1 | (*a*) Indicate the study’s design with a commonly used term in the title or the abstract | 2 |
|  |  | (*b*) Provide in the abstract an informative and balanced summary of what was done and what was found | 2 |
| Introduction | | |  |
| Background/rationale | 2 | Explain the scientific background and rationale for the investigation being reported | 4-5 |
| Objectives | 3 | State specific objectives, including any prespecified hypotheses | 6 |
| Methods | | |  |
| Study design | 4 | Present key elements of study design early in the paper | 6 |
| Setting | 5 | Describe the setting, locations, and relevant dates, including periods of recruitment, exposure, follow-up, and data collection | 6 |
| Participants | 6 | (*a*) Give the eligibility criteria, and the sources and methods of selection of participants. Describe methods of follow-up | 6 |
|  |  | (*b*) For matched studies, give matching criteria and number of exposed and unexposed | NA |
| Variables | 7 | Clearly define all outcomes, exposures, predictors, potential confounders, and effect modifiers. Give diagnostic criteria, if applicable | 6-8 |
| Data sources/ measurement | 8* | For each variable of interest, give sources of data and details of methods of assessment (measurement). Describe comparability of assessment methods if there is more than one group | *6-8* |
| Bias | 9 | Describe any efforts to address potential sources of bias | 6 |
| Study size | 10 | Explain how the study size was arrived at | 6 |
| Quantitative variables | 11 | Explain how quantitative variables were handled in the analyses. If applicable, describe which groupings were chosen and why | 7-9 |
| Statistical methods | 12 | (*a*) Describe all statistical methods, including those used to control for confounding | 8-9 |
|  |  | (*b*) Describe any methods used to examine subgroups and interactions | 9 |
|  |  | (*c*) Explain how missing data were addressed | 9 |
|  |  | (*d*) If applicable, explain how loss to follow-up was addressed | NA |
|  |  | (*e*) Describe any sensitivity analyses | 9 |
| Results | | |  |
| Participants | 13* | (a) Report numbers of individuals at each stage of study—eg numbers potentially eligible, examined for eligibility, confirmed eligible, included in the study, completing follow-up, and analysed | 10 |
|  |  | (b) Give reasons for non-participation at each stage | 10 |
|  |  | (c) Consider use of a flow diagram | Figure 1 |
| Descriptive data | 14* | (a) Give characteristics of study participants (eg demographic, clinical, social) and information on exposures and potential confounders | 10 |
|  |  | (b) Indicate number of participants with missing data for each variable of interest | Table 1 |
|  |  | (c) Summarise follow-up time (eg, average and total amount) | NA |
| Outcome data | 15* | Report numbers of outcome events or summary measures over time | 10 |
| Main results | 16 | (*a*) Give unadjusted estimates and, if applicable, confounder-adjusted estimates and their precision (eg, 95% confidence interval). Make clear which confounders were adjusted for and why they were included | 10 |
|  |  | (*b*) Report category boundaries when continuous variables were categorized | NA |
|  |  | (*c*) If relevant, consider translating estimates of relative risk into absolute risk for a meaningful time period | NA |
| Other analyses | 17 | Report other analyses done—eg analyses of subgroups and interactions, and sensitivity analyses | 11-12 |
| Discussion | | |  |
| Key results | 18 | Summarise key results with reference to study objectives | 10-11 |
| Limitations | 19 | Discuss limitations of the study, taking into account sources of potential bias or imprecision. Discuss both direction and magnitude of any potential bias | 14-15 |
| Interpretation | 20 | Give a cautious overall interpretation of results considering objectives, limitations, multiplicity of analyses, results from similar studies, and other relevant evidence | 12-16 |
| Generalisability | 21 | Discuss the generalisability (external validity) of the study results | 15 |
| Other information | | |  |
| Funding | 22 | Give the source of funding and the role of the funders for the present study and, if applicable, for the original study on which the present article is based | 16 |

*Give information separately for exposed and unexposed groups.

**Note:** An Explanation and Elaboration article discusses each checklist item and gives methodological background and published examples of transparent reporting. The STROBE checklist is best used in conjunction with this article (freely available on the Web sites of PLoS Medicine at http://www.plosmedicine.org/, Annals of Internal Medicine at http://www.annals.org/, and Epidemiology at http://www.epidem.com/). Information on the STROBE Initiative is available at http://www.strobe-statement.org.
